# Editorial: Applications of next generation sequencing (NGS) technologies to decipher the oral microbiome in systemic health and disease, volume II

**DOI:** 10.3389/fcimb.2024.1532762

**Published:** 2024-12-11

**Authors:** Thuy Do, Dongmei Deng, Naile Dame-Teixeira

**Affiliations:** ^1^ Division of Oral Biology, School of Dentistry, University of Leeds, Leeds, United Kingdom; ^2^ Department of Preventive Dentistry, Academic Centre for Dentistry Amsterdam (ACTA), University of Amsterdam and The Vrije Universiteit (VU), Amsterdam, Netherlands; ^3^ Department of Dentistry, School of Health Sciences, University of Brasília, Brasilia, Brazil

**Keywords:** microbiome, dysbiosis, systemic health, NGS - next generation sequencing, oral - general health

Building on the success of our first Research Topic, we present the second volume of *Applications of NGS Technologies to Decipher the Oral Microbiome in Systemic Health and Disease*. Featuring 5 new contributions, of which 1 perspective and 4 original research articles, this volume continues to explore the cutting-edge research on oral health and systemic diseases.

Our first volume collated 17 insightful papers exploring the use of microbial meta-omics data to better understand the complex biological context of health and disease. These contributions provided a comprehensive overview of the current state of knowledge and future directions in the field of oral microbiome research using NGS technologies. NGS technologies hold immense potential for developing strategies to modulate the oral microbiome for improved overall health. A key theme emerging from these studies is the significant impact of systemic conditions, such as hypertension and hyperglycaemia, and dysbiosis in distant body sites, like the gut, on the oral microbiota via the oral-gut axis. This intricate connection between the oral cavity and the gut warrants significant attention. In this volume, Lin et al. investigated the presence of oral bacteria in the gut of patients without any history of intestinal disorders. Their research give compelling evidence for the transmission of oral taxa, commonly residing on the tongue dorsum, to the rectum. Notably, they discovered that the translocation of oral bacteria to the rectum was significantly more prevalent in participants with advanced age, hypertension, and those utilizing proton pump inhibitors.

This volume also features an interesting perspective by Filardo et al. that explores the human microbiome as a “hidden organ”, with important contribution to host functions and significant influence on human health. The review provides insights into human microbiome profiling, describing various metagenomic methods, including 16S rRNA gene sequencing and its associated biases (partial or full-length of the 16S rRNA gene) for the accurate identification of bacteria to the species level, as well as biases associated with different sequencing platforms, that may lead to the underestimation of the biodiversity of the microbiota being studied.

For a more comprehensive picture, shotgun metagenomics offers a deeper taxonomic and functional characterisation of the microbiome (de Cena et al., 2021; Kifle et al., 2024). While first and second-generation sequencing technologies revolutionised the field by enabling higher-resolution analyses, further advancements have brought about third-generation platforms like PacBio and Oxford Nanopore Technologies. These platforms offer long-read sequencing, which, when combined with short-read methods (although admittedly more expensive), can significantly improve coverage and assembly performance, facilitating the detection of low-abundance species. This combined approach would provide a more thorough characterisation of the microbiota and offer a clearer picture of microbial interactions during the transition to dysbiosis (de Cena et al., 2021). However, the high cost of combining these sequencing approaches remains a significant obstacle. This cost disparity leads to a major limitation in the current data landscape. Microbiome research data is often predominantly derived from wealthier countries like the USA and Europe, due to their greater research funding resources. This lack of global representation limits our understanding of how factors like cultural practices, dietary habits, and ethnic backgrounds influence the human oral microbiome (Dame-Teixeira et al., 2021). Investing in cost-effective sequencing methods and fostering research collaborations across diverse populations are crucial steps in overcoming these limitations. By addressing these challenges, we can gain a more complete understanding of the human microbiome’s role in health and disease on a global scale.

Additionally, Filardo et al. highlight the importance of integrating multiple layers of information (multi-omics) in future studies to fully understand the complex relationship between microbiomes and their host. However, the accumulation of larger, more complex microbiome datasets poses significant challenges, requiring specialised computing and bioinformatic skills. To overcome these hurdles and enhance our analyses, researchers are turning to machine learning algorithms to explore complex data and uncover intricate interactions between microbes and their host.

The paper by Chen et al. is an example of the application of machine learning as a diagnostic tool for distinguishing and predicting osteosarcoma from oral microbiota profiles. The authors propose the use of a random forest classifier to uncover distinct microbial signatures within the tongue microbiota of patients with and without osteosarcoma. Specifically, the higher abundance of members of the *Bacteroidota* and *Selenomonas* phyla in osteosarcoma samples allowed the identification of specific operational taxonomic units (OTUs) as potential oral microbial markers.

To better understand the role of the human microbiome in pathogenesis, we must delve deeper into the intricate network of interactions, encompassing microbial-microbial and host-microbial relationships. This network is influenced by small molecules and metabolites produced by both host and microbial metabolisms, as highlighted by (Filardo et al.). Other important genetic, epigenetic and host immune factors should also be taken into consideration in the microbial etiopathogenesis (Hernandez Martinez et al.). Although multi-omics approach can provide the integration of various biological aspects, to fully harness the potential of this approach, we need to develop more sophisticated analysis tools and standardise data collection practices. The paper by Dorst et al. provides such attempt. The authors emphasise the importance of data sharing, which facilitates collaboration, enables new discoveries, and saves time and resources. However, challenges such as incomplete metadata, incompatible software, and lack of standardisation hinder data reuse. Implementing “FAIR” principles can address these issues by ensuring data is “findable, accessible, interoperable, and reusable”. This can lead to more efficient data handling, faster insights, and reduced research costs, through the deployment of a user-friendly framework that promotes inter-disciplinary collaboration. Furthermore, we argue that future studies require much more detailed metadata. Research on the oral microbiome and systemic health should include comprehensive data on the donor’s oral health, including periodontal disease and caries, as well as well-characterised and reproducible disease diagnoses. However, there are some concerns regarding General Data Protection Regulation (GDPR) for data privacy with regards to patients’ data information, which may limit such implementation in healthcare research.

In conclusion, the oral microbiome undeniably impacts overall human health. A deeper understanding of host-microbe interactions can inform targeted strategies for disease prevention and treatment. This knowledge can guide the development of novel preventive strategies through microbiome modulation. Moreover, achieving predictive health and disease models for personalised therapies focused on restoring a healthy microbiome will necessitate collaboration between microbiologists and clinicians to strengthen the connection between biological and clinical characteristics.
